# Chemotherapy and Other

**DOI:** 10.1080/135771402753667682

**Published:** 2002-05


### CHEMOTHERAPY AND OTHER

### ORAL PRESENTATIONS

### E98 Comparison of Doxorubicin Versus VP16-Ifosfamide in Addition of High Dose Methotrexate as Pre-operative Chemotherapy in Osteosarcomas. A Randomized Trial by the French Society of Pediatric Oncology

L.B. Brugieres[1], C. Kalifa[1], J.M. Guinebretiere[1], J.C. Gentet[2], T. Philip[3], N. Dupouy[1], N. Bellon[1], C. Rodary[1]
[1]*Institut Gustave Roussy, Villejuif, France,* [2]*Hospital La Timone, Marseille, France,* [3]*Centre LTon BTrard, Lyon, France*

*Objectives:* Long term cardiac toxicity of Doxorubicin led the French Society Pediatric Oncology to conduct a trial to compare the efficacy of a combination of HDMTX and Doxorubicin with a combination of HDMTX with VP16-Ifosfamide (VP-Ifo). The choice of VP-Ifo was the based on the good response rate obtained in a previous phase II study of this combination in pre-treated osteosarcoma.
*Methods:* All patients, less than 20 years, newly diagnosed with non metastatic limb osteosarcoma could be included in the trial and randomized to receive 7 courses of HDMTX 12 g/m$^2$ in combination either with Doxo 70 mg/m$^2$ (2 courses) in arm S or with VP16 75 mg/m$^2$/dx4d – Ifosfamide 3 g/m$^2$/dx4d (2 courses) in arm N. Postoperative chemotherapy was adapted to the histological response. The main criteria was the proportion of good histological responses (<5% viable cells).
*Results:* 227 localized limb high-grade OS pts aged 3 to 19 years have been randomized; 2 pts have been excluded from analysis because of wrong diagnosis. Surgery was conservative in 212 pts and radical in 13. Compliance to pre-operative chemotherapy was good as 85% and 91% of the pts received at least 6 courses of HD MTX and 2 courses of Doxo or VP-Ifo respectively. Granulopenia < 0.5×109/l occurred in 58% of the courses in arm S and 77% in arm N. Good histological response was observed in 43/113 pts (42%) in arm S and 61/112 (54%) in arm N. The observed difference between the percentage of good responders between the 2 arms is 12% (p=0.05). For the whole population, the EFS3y is 75% and OS3y 84%.
*Conclusion:* the association of HD MTX and VP-Ifo produces a significantly higher proportion of good histological response and will be considered for further studies.

### E99 Gene Therapy of Osteosarcoma: Targeting Adenoviruses Towards Integrins Increases Transduction Efficiency and Tumour Cell Kill in vitro and in vivo

M.A. Witlox, V.W. Van Beusechem, A. De Gast, G.A. Schaap, H. Bras, P. Van Diest, R. Alemany, D.T. Curiel, H.M. Pinedo, W.R. Gerritsen, P.I.J.M. Wuisman
*VU University Medical Centre, Amsterdam, The Netherlands*

Conditionally Replicative Adenoviruses (CRAds) are attractive agents for Osteosarcoma (OS) gene-therapy. However, adenovirus infection of OS is limited due to low levels of the coxsackie-adenovirus receptor (CAR). Targeting adenoviruses to another receptor highly expressed on OS could overcome this limitation. We found that CAR expression was low or absent on OS. Integrins alpha-v-beta-3 and alpha-v-beta-5 were found widely expressed on primaryOS as well as on vascular endothelial cells within the tumour. We hypothesized that integrins would be valuable targets to redirect adenoviruses. This hypothesis was tested on four OS cell lines (MNNG-HOS, MG-63, CAL 72, SaOs-2) and a panel of primary OS cell cultures. The integrins were targeted through insertion of an integrin-binding RGD motif into the HI loop of the adenovirus fiber. Targeting enhanced gene transfer efficiency up to 100 fold. Tumour specificity of a CRAd can be achieved via deletions in viral genes. In this study the CRAd Addelta24 was used. This CRAd selectively replicates in cells with an abnormal Rb pathway. Targeting Addelta24 towards integrins (Addelta24RGD) showed to be most efficient in killing primary OS tumour cells in vitro. Twelve days post infection at an MOI of 0,1 Addelta24RGD cells were all dead, while Addelta24 infected cells were unaffected. Moreover, Addelta24RGD injected into human primary OS tumours (volume 200mm3) growing on the flanks of nude mice induced a significant tumour growth delay. PBS treated control tumours reached 5 times the initial volume within 10 days. Addelta24RGD treated tumours exhibited a significant growth delay, reaching 1000mm3 after 25 days.
*Conclusion:* This study shows that redirecting adenoviruses towards integrins can efficiently enhance transduction of OS, resulting in more effective killing of OS cells in vitro and in vivo in a fast growing OS. Therefore, this therapeutic approach may be a potential candidate for the future treatment of OS.

### E100 Ewing Tumour (ET) of the Spine – Experience of the Cooperative Ewing's Sarcoma Studies (EI)cess 81–92

S. Ahrens[1], M. Kuhlen[1], M. Paulussen[1], C. Rnbe[2], B. Froehlich[1], J. Dunst[2], W. Winkelmann[2], H. Juergens[1]
[1]*University Children's Hospital, Muenster, Germany,* [2]*University Hospital, Homburg/Saar, Germany*

*Objective:* To determine prognostic factors for spinal ET and to document EFS and local control.
*Methods:* 116 patients (pts) registered within (EI)CESS from 01/81 to 12/99, representing 7.5% of the study population (1549 pts), were analyzed. 63 (54%) were male, the median age was 14.5 years (range: 2 months- 48 years). Initial tumour volume was <100ml in 51 (44%) and 100 ml in 41 (35%). 27 patients (23%) presented with metastases at diagnosis. Local therapy was radiotherapy alone (65%) or surgery ± radiotherapy (31%). EFS was analyzed according to Kaplan and Meier, comparisons by logrank test, and risk factors by Cox models. Competing risks analysis was carried out to assess local and/or systemic failure rates.
*Results:* To date (median time under study 104 months), ten-year EFS was 0.45. Primary metastases (EFS 0.23 versus 0.52 for non-metastatic tumours, p=0.0081), gender (EFS 0.34 for males versus 0.57 for females, p=0.0083), and initial surgery (EFS 0.17 versus 0.48 for patients without initial surgery, p=0.0792) had negative impact on EFS. Tumour volume (EFS 0.36 for 100ml versus 0.53 for smaller tumours, p=0.2105) and local therapy strategy (EFS 0.40 for definitive radiotherapy versus 0.42 for surgery in combination with radiotherapy, p=0.3303) did not significantly influence the prognosis. Multivariate Cox analyses predicted fair outcome for female patients without primary metastases and without primary surgery. The cumulative incidence of local and combined local and systemic failure was 0.28, compared to an incidence rate of 0.33 for systemic metastases and other events. 4/116 patients (3.4%) died of complications during primary treatment, 1/116 patients (0.9%) developed a second malignancy.
*Conclusion:* 54/116 (47%) of patients with ET of the spine survived long term. The local failure rate is increased compared to other primary tumour sites.

### POSTER PRESENTATIONS

### E101 Neoadjuvant Chemotherapy in Limb Soft Tissue Sarcoma: The Significance of C-ERBB-4 Expression

O. Merimsky, J. Issakov, Y. Kollender, I. Schwartz, J. Bickels, G. Flusser, M. Inbar, I. Meller
*Tel Aviv Sourasky Medical Center, Tel Aviv, Israel*

*Purpose:* ErbB-4 is a recently described member of the epidermal growth factor receptor (EGFR) family. Relatively little is known about the expression of erbB-4 in human tumours. In the present study we assessed the possible role of c-erbB-4 expression product as a tissue marker for STS, and its correlation with the response to chemotherapy.
*Methods:* The histological specimen of 29 patients with STS of a limb who had received preoperative doxorubicin-based chemotherapy were studied. The extent of tumour necrosis was evaluated histologically. Paraffin blocks of preoperative incisional biopsy were available for immune staining (avidin-biotin-peroxidase technique) from 29 patients, and blocks of the surgical specimen after pre-operative chemotherapy were available from 27.
*Results:* The objective response rate to preoperative chemotherapy was 34%. Wide resection of the tumour was feasible in 12 patients, marginal resection in 14 cases, amputation in 2 patients with disease progression, and no surgery in one case. The tumour necrosis was above 90% in 9 patients, 60–90% in 12, and less than 60% in 7 patients. An increase in C-erbB-4 expression was more common in cases with no response to chemotherapy, while no change of or decrease in C-erbB-4 was more common in responsive tumours (p=0.004). No correlation could be found between the degree of necrosis or the chemotherapeutic regimen and the change in expression of c-erbB-4. The median DFS was longer for patients with a decrease or no change in expression of C-erbB-4 than for patients with increased expression.
*Discussion:* It is believed that post chemotherapy new expression or no down-regulation of the erbB-4 molecule represents tumour aggressiveness and increased capability of growth and spread.

### E102 The Multicentric Epitheloid Hemangioendothelioma of Bone: A Case Example and Review of the Literature

J. Hardes, G. Gosheger, C. Gebert, W. Winkelmann
*[1]University Hospital of Münster, Münster, Germany*

Epitheloid hemangioendothelioma of bone is a rare primary bone malignancy. Diagnosis can be difficult for physicians who are not experienced with bone neoplasms. We also had difficulties in diagnosis and treatment in one of our patients with a multicentric epitheloid hemangioendothelioma involving the pelvis, left femur and left tibia. We recommend a complete skeletal survey with magnetic resonance imaging because it can reveal previously undetected lesions on conventional radiographs. We performed an extraarticular resection of the hip joint including the spina iliaca anterior inferior, resection of the femur, and amputation of the lower leg. The femur was replaced by a modular endoprosthesis. The patient is provided with an above-knee prosthesis and is able to walk even longer distances with a cane 2 years after surgery. In our opinion it is necessary to perform a wide resection of this tumour in order to treat a patient with a curative intention. Palliative radiotherapy should only be used for a non-resectable tumour or in metatatic disease. Chemotherapy is not a treatment option.

### E103 Familial Coping Patterns with Musculosketeal Tumours Among Arabs in Israel

P. Elad[1], M. Agabaria[2], Y. Yagil[1,2], E. Barmizada[1], I. Meller[1]
*[1]Nat. Unit of Orhtopedic Oncology, Tel Aviv Sourasky Medical Center, Tel Aviv, Israel, [2]Bob Shappell School of Social Work, Tel Aviv, Israel*

*Rationale:* A severe illness, such as cancer, is a challenge not only for the patient but the entire family. It is the family's task to preserve normal life with minimal disruptions while simultaneously taking care of the ill family member. Coping patterns need to be perceived and understood within socio-cultural and ethnic contexts.
*Aims:* This study aimed at learning about coping strategies among Israeli-Arab patients with musculoskeletal cancer and their families.
*Population:* 45 out of 60 Arab patients treated in the National unit of orthopedic oncology between 1996 and 2000, (23 males; 22 females & 78% Muslims; 11% Druse persons & 11% Christians) agreed to be interviewed.
*Methodology:* Interviews used open-ended questions relating to past acts and current perceptions of coping. The two part questionnaire was based on an adaptation and translation of the F-copes questionnaire. Interviews were conducted in Arabic primarily in the patients' homes between March and June 2000.
*Results:* 80% believed that they and their family can cope with difficulties arising from illness. 64% claimed that were they to be ill today they would involve their families with any resulting difficulties. In retrospect 49% did so. Only 22% would and did involve neighbors in their difficulties. 58% stated they would request help from community services only 29% did so during the illness. 40% stated they would consult with a religious leader and when ill 24% did so.
*Conclusion:* The results indicate a belief in the nuclear family as a prime resource for coping and a low use of formal and informal community resources.

### E104 Age Related Variation in the Radiological Appearance of Appendicular Osteosarcoma

A. Saifuddin[1], J.A.S. Pringle[2], J. White[3], T.W.R. Briggs[4], S.R. Cannon[4]
*[1]Royal Nationa Orthopaedic Hospital NHS, London, United Kingdom, [2]Royal National Orthopaedic Hospital NHS, London, United Kingdom, [3]Oldchurch Hospital, London, United Kingdom, [4]Royal National Orthopaedic Hospital NHS, London, United Kingdom*

*Aims:* To determine whether there is any variation in the radiographic appearances of primary appendicular osteosarcoma with age at presentation.
*Methods:* 62 patients with a pathologically proven diagnosis of primary osteosarcoma arising in the appendicular skeleton were retrospectively reviewed. Features including sex, site and various radiological appearances were noted and compared to the age of the patient.
*Results:* The study group consisted of 33 males and 29 females with a mean age of 21.5 years (range 6–79 years). No relationship was identified between bone involved, pattern of bone destruction, size or location of associated soft-tissue mass with age at presentation. The mean age of patients whose tumours extended from the metaphysis into the epiphysis and also to the joint margin was significantly greater than those whose tumours were limited to the metaphyseal region. Degree of matrix mineralization was less in those patients under 15 years or over 25 years. This finding almost reached statistical significance. Absence of periosteal reaction and Codmans triangle was also significantly associated with an older age of presentation.

*Conclusion:* Some features of the radiological appearance of primary appendicular osteosarcoma vary according to the age of patient at presentation and may result in an erroneous radiological diagnosis.

**E105 Acute Pancreatitis after Ifosfamide. A Rare but Heavy Complication**

N. Delepine, S. Alkallaf, B. Markowska, H. Cornille, I.M. Bigirimana, G. Delepine
*Avicenne hospital, Bobigny, France*

Ifosfamide is one of the most important drug in treatment of soft and bone tissue tumours. We use it from 1985 and around 150 courses a year are performed in our unit. We saw only two times a pancreatitis following continuous infusion of ifosfamide. In 1994 June, a 35-year-old female with fibrosarcoma presented a symptomatic pancreatitis following a ifosfamide-vincristine-actinomycine combination. Ifosfamide was given continuously at the dosage of 3 g/sqm/day, 2 days. The pain was important during 3 weeks and led to stop ifosfamide therapy in this patient. Recently, in 2002 January, we treated a 17-year-old girl with relapsed rhabdomyosarcoma. She received ifosfamide as a single drug at the dose of 3 g/sqm/day (theoretically five days) with corticosteroids and antiemetics. At the beginning of the third day, she presented dramatically abdominal pain, vomiting, was doubled up. Symptomatic treatment by antispasmodics was effective associated with stop of chemotherapy. Biologic examination showed increased amylasemia (725 IU the first day, 1077 the second -normal <82-) and increased amylasuria 2208 IU (Normal <750), increased lipasemia 3478 IU (normal <300). CTScan showed oedematous pancreas. Resolution of clinical and biological symptoms was complete in 3 days. We founded only two reports of acute pancreatitis associated with ifosfamide in the literature. So, we think important to monitor routinely serum amylase levels during ifosfamide infusion, to approach the true incidence of this complication and to be able to determine the necessity of stopping definitively the use of this drug in patients presenting one time signs of pancreatitis. The role of corticoids in the apparition of pancreatitis must be evoked and leads to avoid, as soon as possible, the association of corticoids with ifosfamide.

**E106 Survival and Prognostic Factors in Osteosarcoma, a Retrospective Analysis**

J.A.M. Bramer[1], M.J.J. Veth[1], J. Bras[1], J. De Kraker[2], J.W. Vd Eijken[1], G.R. Schaap[1]
[1]*Academic Medical Centre, Ouderkerk A.D. Amstel, The Netherlands,* [2]*Emma Kinderziekenhuis, Amsterdam, The Netherlands*

*Introduction:* Survival in osteosarcoma varies from 50–80%. In literature response to chemotherapy is the most important prognostic factor. Other described factors are gender, age, tumour-location and -volume, stage, alkaline phosphataselevel and delay to treatment. The value of these remains however controversial. Goal of this study was to assess survival in osteosarcoma patients treated in our institute, and to evaluate the value of abovementioned factors.
*Methods:* Retrospective study of all patients treated in AMC/EKZ for high-grade extremity osteosarcoma (1985–1996), with minimally 5 years follow up. In case-sheet survey patient- and tumour characteristics, treatment and results were investigated. Prognostic factors were related to survival.
*Results:* 60 patients were treated, 5 lost to follow-up. The 5 years survival was 62%. There was no gender related difference in survival. Patients aged 10–15 and 15–20 seemed to have worse survival rates (48% and 57%) then other age groups (73%), this difference however was not significant. Tumour location was mostly (44×) around the knee, but had no correlation with survival. There were insufficient data to evaluate prognostic value of tumour volume. Stage (Enneking) was mostly II-B (survival 65%). All 6 patients with stage III deceased. A clear correlation occurred between Alkaline Phosphatase and survival (from 82% with AF<200 to 25% with AF>500). To a lesser extent this was found for response to chemotherapy: 82% in good, 56% in bad responders (EOI standard). No significant correlation occurred between survival and delay.
*Conclusion:* In this patient group survival was comparable with literature. Gender had no prognostic value. Stage and Alkaline Phosphatase level correlated well with survival. To a lesser extent this was true for response to chemotherapy. More accurate parameters and a weighted score-system would improve prognostication.

**E107 Serum Markers of Bone Turnover in Patients with Osteosarcoma of the Extremity Treated with Primary Chemotherapy**

S. Ferrari, L. Pratelli, R. Turrini, S. Tedioli, L. Loro, C. Forni, S. Giacomini, G. Bacci
*Istituti Ortopedici Rizzoli, Bologna, Italy*

*Objective:* To assess bone turnover in patients with osteosarcoma treated with primary chemotherapy.
*Methods:* 46 patients (median age 17, 7–42) with osteosarcoma of the extremity entered the study. Blood samples were taken at the baseline and before surgery. Serum concentration of bone specific alkaline phosphatase (BALP), and osteocalcin (OC) (markers of bone formation), and carboxy-terminal telopeptide of type I collagen (ICTP) (marker of bone resorption) were measured by immunometric essay and expressed as ng/mL, μg/L, ng/mL respectively. All patients underwent surgery after good clinical and radiologic response. With a median follow up of 26 months, 31 patients are continuously free of disease.
*Results:* At the baseline, serum concentration of BALP, OC, and ICTP was 48.1±38, 38.2±22, and 1.05±0.5 respectively, and it changed to 16±9 (p=0.0001) 21±9.7 (p=0.0001), and 1.4±0.5 (p=0.0002) after primary chemotherapy. Patients with a good histologic response showed higher baseline BALP serum level compared to those with a poor response (40±42 vs 28±17, p=0.008) whereas no differences were found in the post primary chemotherapy samples according to the histologic response. Patients who relapsed had higher baseline level of BALP (69 vs 39, p=0.008), OC (47.6 vs 34.1, p=0.06), and ICTP (1.26 vs 0.9, p=0.09). After primary chemotherapy, relapsing patients showed persistent higher level of OC (25.9 vs 18.7, p=0.05) and ICTP (1.62 vs 1.28, p=0.09), but no difference for BALP (17.9 vs 15.1).
*Conclusion:* In patients with osteosarcoma a significant reduction of bone formation and an increase of bone resorption is induced by primary chemotherapy A higher bone remodelling activity was observed in patients who relapsed suggesting a relation between tumour aggressiveness and bone turnover.

**E108 Outcome of Flat Bone Sarcomas (other than Ewing's) in Children and Adolescents/a Study of 25 Cases**

V. Minard-Colin, C. Kalifa, J.M. Guinebretiere, L. Brugieres, J. Dubousset, J.L. Habrand, G. Vassal, O. Hartmann
*Institut Gustave Roussy, Villejuif, France*

*Objective:* we analysed the clinical features and outcome of young patients with non-Ewing's flat bone sarcoma treated during the era of contemporary chemotherapy.

*Methods:* The characteristics and outcome of 25 patients (15 males and 10 females) with primary or radiation-related flat bone sarcoma treated in the Pediatrics Department at the Institut Gustave Roussy from 1981 to 1999 were reviewed.
*Results:* Twenty patients had osteosarcoma, four chondrosarcoma and one malignant fibrous histiocytoma. Age at diagnosis ranged from 2 to 23 years (median, 15 years). Nine tumours were located in the craniofacial bones, eleven in the pelvis and five in flat bones at other sites. Four patients had metastatic disease at diagnosis. Radiation-associated flat bone osteosarcoma was diagnosed in 10/25 cases. The median time between radiotherapy and the diagnosis of secondary osteosarcoma was 7 years. The projected overall survival and event-free survival (EFS) rates at 5 years were 45.1% (+/- 19%) and 34.3% (+/- 18%) for all 25 patients. The EFS rate of patients with a second bone sarcoma was similar to that of patients with de novo flat bone sarcoma (p=0.1). The aim of treatment was curative for 24 patients, 23 of whom were treated with intensive chemotherapy regimens and 19 with surgery. Significant adverse prognostic factors on survival included incomplete surgical resection (p=0.001) and used of regimens without pre and postoperative chemotherapy (p=0.007). Nine of the 25 patients were treated with pre and postoperative chemotheray and complete surgical resection. Among them, eight are alived with no disease.
*Conclusion:* Radical surgical resection is the overriding prognostic factor for flat bone sarcomas in young patients. Nevertheless, our Results suggest a more favorable outcome since the advent of intensive chemotherapy.

**E109 CD44 Is a Prognostic Marker in Soft Tissue Sarcoma**

M. Peiper, T. Sato, W.T. Knoefel, D. Zurakowski, J.R. Izbicki
*University Hospital Hamburg-Eppendorf, Hamburg, Germany*

*Background:* The expression of CD44 has been identified as prognostic factor in several malignant diseases. However, only few data exist correlating CD44 expression in soft tissue sarcoma with subsequent tumour progression or recurrence. The purpose of this study was to investigate the clinical significance of CD44s in adult soft tissue sarcoma (STS).
*Methods:* Tumour specimens of 62 patients with STS were evaluated regarding CD44s expression using immunohistochemistry. The significance of the proposed prognostic indicators was evaluated in relation to survival and local recurrence.
*Results and Conclusion:* Of 62 analysed specimens, 49 tumours were CD44s positive compared to 13 CD44s negative tumours. Kaplan-Meier survival analysis indicated significantly better survival among patients whose tumour was CD44s positive (P=0.015). Variables predictive of longer survival included resection quality (R0, P<0.01) and tumour size (T1, P=0.02). CD44s expression correlates with prognosis of soft tissue sarcomas. CD44s may, therefore, play a pathogenetic role in tumour progression Determining the expression of CD44s in primary STS could be a valuable tool for selecting patients for further adjuvant treatment. Nevertheless, radical resection at initial surgery plays the pivotal role in soft tissue sarcoma treatment.

**E110 Treatment of Advanced Pelvic Osteosarcoma with Chemotherapy Directly Inside the Tumour. Case Report.**

N. Delepine, S. Alkallaf, B. Markowska, H. Cornille,
I.M. Bigirimana, G. Delepine
*Avicenne hospital, Bobigny, France*

*Introduction:* Very large tumours of pelvis may seem inoperable and are often treated by palliative radiotherapy and chemotherapy. We present here a case with intratumoural chemotherapy and mid-term result.
*Case report:* This girl, 16 years old, came from Africa in 2000 July, for a huge tumour of pelvis after 2 years of pelvic pain. A needle biopsy confirmed the diagnosis of high-grade osteosarcoma. Initial screening showed a huge tumour (15 cm×12×10) developed from the right acetabulum, with compression of bladder and both ureters. The dilatation of kidney cavities led to perform a bilateral ureterostomy. Conventional chemotherapy didn't obtain any response and the patient was sent in a palliative care unit after 2 months of an ineffective treatment. In October 2000, she was referred for treatment in our unit. We started by intratumoural debulking and put inside the tumour cavity a chemotherapy infusion catheter. Following 3 courses of intratumoural chemotherapy (CDDP 25 mg) associated with systemic chemotherapy, new debulking surgery was performed and the normal urinary tract could be repaired. Both intra-tumour and intravenous chemotherapy were continued and in October 2001, the last tumour residual was removed and a composite acetabular reconstruction performed.
*Result:* In January 2002, the patient is in complete remission. She can walk, go to school and live normally. The hip function rate according to Enneking's criteria is excellent.
*Conclusion:* Advanced sarcoma can sometimes benefit of an individualized treatment including intratumoural chemotherapy.

**E111 Ewing Sarcoma of the Mobile Spine – Report on 14 Cases**

A. Gasbarrini[1], S. Bandiera[1], F. de Iure[1], S. Ferrari[2], P. Bacchini[2], A. Barbieri[3], P. Picci[2], L. Boriani[1], S. Boriani[1]
[1]*Dept. of Orthopedics and Traumatology, Maggiore Hospital, Bologna, Italy,* [2]*Istituti Ortopedici Rizzoli, Bologna, Italy,* [3]*S. Orsola Hospital, Bologna, Italy*

*Study Design:* Fourteen cases of Ewing sarcoma of the mobile spine were retrospectively reviewed.
*Objectives:* To evaluate the role of surgical and non-surgical treatment of Ewing sarcoma of the spine. Summary of Background data: Ewing sarcoma involved very rarely the spine; frequently occurred during second decade of age. The course of the disease depends on the aggressiveness of the tumour, as well as the treatment. Although the anatomic-characteristics of the spine, preoperative-chemo and radiation-therapy combined to a wide excision seems to be an effective treatment.
*Methods:* All charts, radiographs and images were reviewed. The composite information provided by this review allowed for oncologic and surgical staging of these cases. 1 patient underwent curettage, 5 underwent en bloc excision. All those patients were submitted to chemo and or radiation therapy too. Eight patients underwent decompressive laminectomy combined to adjuvant therapies or chemo and radiation therapy only (palliative treatment).
*Results:* On the series of palliative treatment, six patients submitted to palliative treatment were found dead at final follow up evaluation, while only 2 were alive without evidence of disease. One patient submitted to intralesional excision were found alive at final follow up evaluation. In en bloc excision series 3 patients were found alive and disease free, 1 patient was alive and 2 cases were dead at final follow up evaluation. Only one local recurrence was diagnosed and treated by an intralesional excision and radiation therapy.
*Conclusion:* If confirmed on larger series, pre and post-operative adjuvant therapies combined to a wide excision of the tumour seems to be the first treatment option for spinal osteosarcoma. Diagnosis must be certain, based on pathognomonic radiographic pattern and histologic study. In case of neurologic involvement and pathologic fracture, complete intralesional excision and post-operative adjuvant therapy would be the therapy of choice.

**E112 Osteosarcoma of the Mobile Spine – Report of 16 Cases**

S. Boriani[1], A. Gasbarrini[1], S. Bandiera[1], F. de Iure[1], L. Boriani[1], F. Bertoni[2], P. Picci[2], G. Bacci[2]
*[1]Maggiore Hospital, Bologna, Italy, [2]Istituti Ortopedici Rizzoli, Bologna, Italy*

*Study Design:* Sixteen cases of osteosarcoma of the mobile spine were retrospectively reviewed.
*Objectives:* To evaluate the role of surgical and non-surgical-treatment of osteosarcoma of the spine.
*Summary of Background data:* Osteosarcoma involved very rarely the spine; frequently occurred in young-adult patients. The course of the disease depends on the aggressiveness of the tumour, as well as the treatment. Although the anatomic-characteristics of the spine, pre-operative chemo-therapy combined to a wide excision and post-operative chemo and radiation therapy seems to be an effective treatment.
*Methods:* All charts, radiographs and images were reviewed. The composite information provided by this review allowed for oncologic and surgical staging of these cases. Six patients underwent curettage, 6 underwent en bloc excision, one patient underwent palliative decompression. All those patients were submitted to chemo and or radiation therapy too. Three patients received radiation therapy combined to chemotherapy alone.
*Results:* Three patients submitted to intralesional-excision were found alive and disease free at final follow-up-evaluation. At mean time three patients were dead. In en bloc excision series 2 patients were found alive and disease free, 1 patient was alive, but suffering by lung-metastases and 3 patients were dead at final follow-up evaluation. All patients submitted to adjuvant therapy only were found dead. One only patient was submitted to a palliative surgery and was found alive at final follow up evaluation while is already submitted to adjuvant therapies.
*Conclusion:* If confirmed on larger series, pre and post-operative adjuvant therapies combined to a wide excision of the tumour and post-operative radiation therapy seems to be the first treatment option for spinal osteosarcoma. Diagnosis must be certain, based on pathognomonic radiographic pattern and histologic study. In case of neurologic involvement and pathologic fracture, complete intralesional excision and post-operative adjuvant therapy would be the therapy of choice.

**E113 Radiofrequency Ablation for Locally Recurrent Sarcoma**

J.B. Cobb, M.A. Hall-Craggs, A.E. Walker, J.S. Whelan, J.D. Witt, A. Cassoni, W.R. Lees
*University College London Hospitals, London, United Kingdom*

Local recurrence of sarcoma poses one of the most difficult problems. A small recurrence may trigger an operation that is more radical than the first, with less chance of cure, because the it is almost always much more difficult to obtain good margins second time around. After learning from the group in Leiden of local therapies for benign disease, and having used percutaneous modalities in the management of liver and lung metastases, we obtained ethics committee approval to treat patients with relapsed sarcoma. Conventional treatment modalities had been rejected by the multidisciplinary meeting on the grounds of likely ineffectiveness and high morbidity. We report on the first 8 cases entered into this study.
*Methods:* 8 cases were treated between 1998 and 2001. The pathological diagnoses were: 3 Chondrosarcoma, 2 Malignant Fibrous Histiocytoma, 1 Chordoma, 1 ewings and 1 Fibromatosis. The patients were aged between 30 and 82 years. All tumours were recurrent, having relapsed after surgery between 1 and 4 times. RF was used on 3 occasions in one patient, and twice in two locations in one patient. Interstitial Laser Photocoagulation and Photodynamic therapy were also used once. Follow up by has been performed by serial scanning with MRI, or CT or both.
*Results:* local control without surgery : 4/6; local effect enabling surgical success: 2/2. complications: 1 fistula, 1 skin breakdown, 2 neurological damage. All were within an irradiated field.
*Conclusion:* RF provides a highly effective alternative to surgery for early recurrent sarcoma where surgery may be difficult or dangerous. Collateral damage is still a problem in irradiated tissue.

**E114 Skeletal and Soft Tissue Ewing's Sarcoma: Is there a Difference**

I.W. Carmichael, R. Grimer
*Royal Orthopaedic Hospital, Birmingham, United Kingdom*

Some tumours behave differently depending on their site of origin. Ewing's-sarcoma of soft-tissues is a rare tumour, and discussions on treatment are scanty. We set out to compare the mortality and treatment response between Ewing's – We performed a retrospective review of our database. Between 1975 and March 2001, we identified 402 patients with a definite diagnosis of Ewing's-group-sarcoma. There were 349 with skeletal-tumours and 53 with soft-tissue. The same multidisciplinary-team reviewed all patients. All tumours were treated with the most up to date chemotherapy-regime available at that time. Surgery and radiotherapy were determined on an individual basis. There were no differences between the groups with regard treatment options or modalities. There was a significant difference between the ages of the 2 populations (mean for skeletal 18 years, mean for soft tissue 27 years, t' test $p<0.0001$), the number of Pnet-subtypes in each group (skeletal 28/349 (8%), soft tissue 11/53 (21%), chi square $p=0.003$), and Enneking-stage (2% of skeletal and 26% of soft tissue were 2a (expected 6%), chi square $p<0.0001$). However, there was no significant difference in either the overall-survival (skeletal 58%, soft tissue 53%, $p>0.2$), or overall disease free survival (skeletal 44%, soft tissue 45%). Similarly, there was no significant difference in the overall-rate of metastases (skeletal 54%, soft tissue 51%, $p>0.2$), rate of metastases at diagnosis (skeletal 20%, soft tissue 19%), or in mortality relating to Enneking-stage or chemoresponse. Although the populations were significantly different, the disease process as defined by mortality and treatment response was exactly the same.
*Conclusion:* Skeletal and Soft-tissue-Ewings-sarcoma are the same disease manifest in different populations, depending on site of origin. It would also seem that treatment-Methods found to be affective in 1 group will be affective across both groups.

**E115 Ewing's Sarcoma and Primary Neuro-Ectodermal Tumour of the Soft Tissue: Epidemiology, Prognosis and Results of Combined Treatment**

N. Fabbri, S. Ferrari, E. Guerra, P. Picci, M. Mercuri
*Istituti Ortopedici Rizzoli, Bologna, Italy*

*Introduction:* Soft tissue Ewing's sarcoma (ES) and Primary Neuro-Ectodermal Tumour (PNET) are rare high-grade malignant neoplasms presenting very similar features, considered strictly related by most authors. Literature is scant and Results of large series are lacking. Purpose of this study was to analyse epidemiology, risk factors, and Results of different treatment modalities.
*Methods:* A retrospective study was undertaken at the authors' Institution. Epidemiology, clinico-pathologic features, staging, tumour volume, laboratory tests and different treatment regimens were investigated.
*Results:* Forty-three cases of soft tissue ES-PNET were identified (23 males, 20 females; average age 29). Most common site was the lower extremity (26 cases); 40 cases were admitted for treatment, 3 were sent for consultation. In 8 of the 40 cases admitted for treatment there was evidence of metastatic disease. Average tumour largest dimension was 11 cm (range: 3–28). Different treatment regimens combining chemotherapy, radiotherapy and/or surgery were used over a period of 20 years. Minimum follow-up of 2 years was available in 37 patients. Overall disease-free survival (DFS) is 57% (21 of 37) while continuously disease-free (CDF) rate is 49% (18 of 37). Seven of the 8 patients with metastatic disease at presentation died. A subgroup of 21 patients with localised disease were homogeneously treated with contemporary chemotherapy and surgery. At a minimum follow-up of 2 years, DFS rate for localised disease in this group is 76% (16 of 21).
*Conclusion:* Soft tissue ES-PNET is rare, most common in the lower extremity during the third decade. Best survival rate was associated with the combination of chemotherapy and surgery.

**E116 Malignancy in Giant Cell Tumour of Bone**

E.L. Staals, F. Bertoni, P. Bacchini
*Istituti Ortopedici Rizzoli, Bologna, Italy*

*Objective:* Malignancies in giant cell tumour of bone are rare events, in some cases difficult to recognize. The purpose of this study was to define the clinicopathologic and histologic features of these lesions.
*Methods:* We reviewed all cases of primary (PMGCT) and secondary (SMGCT) malignancies in giant cell tumour of bone seen at our institution. PMGCT is a high-grade sarcoma juxtaposed to areas of benign giant cell tumour (GCT). SMGCT is a high-grade sarcoma arising on the localization of a GCT previously treated by surgery or radiotherapy.
*Results:* There were 5 PMGCT's and 12 SMGCT's. Half of the SMGCT's occurred after radiotherapy. Age in patients with PMGCT ranged from 20 to 68 (median 62), in those with SMGCT from 30 to 77 (median 40). In both PMGCT and SMGCT the most frequently involved sites were the long bones around the knee. Latent time between diagnosis of GCT and diagnosis of SMGCT was on average 9 years (range 3–15) in patients subjected to radiotherapy and 18 (range 7–28) in those who had spontaneous transformation. The high-grade sarcomas in PMGCT were osteosarcomas (4) and malignant fibrous histiocytoma (1). The SMGCT's were osteosarcomas (9), fibrosarcomas (2) and malignant fibrous histiocytoma (1). Two patients with PMGCT had lung metastasis, one died after 8 months, one is alive at 40 months. The other patients with PMGCT have no evidence of disease (NED) 2, 15 and 161 months after the diagnosis. All patients with post-radiation SMGCT died (after 5, 5, 9, 13, 20 and 148 months). Two of the patients with spontaneous SMGCT died of metastasis at 2 and 3 months; one died of unrelated cause at 122 months; the other three have NED at 32, 113 and 123 months after the diagnosis.
*Conclusion:* PMGCT and SMGCT are rare high-grade sarcomas with poor prognoses.

**E117 Solitary Fibrous Tumour of Extrapleural Site. 3 Cases with Bone Involvement (Sacrum, Pubic Bone, Radius).**

A.R. Von Hochstetter[1], G.U. Exner[2]
*[1]Pathologie Institut Enge, Zurich, Switzerland, [2]University of Zurich, Orthopaedie, Zurich, Switzerland*

*Introduction:* Solitary fibrous tumour (SFT) of extrapleural site is a pathologic entity that has been recognized only recently. We wish to report 3 consecutive cases with bone involvement to draw the attention to this diagnosis in musculoskeletal tumours.
*Methods:* Three patients have been diagnosed to have SFTES and were treated by wide resection. Patient characteristics are: Ages of the patients were 28, 47 and 49 years; duration of symptoms varied from 2 months to 4 years; localizations were the Sacrum, pubic bone and the radius. Needle biopsies in the three patients were suggestive of SFT and wide resections were performed because of the uncertain dignity. At present no recurrence or metastases were observed at follow-up for 30 to 36 months.
*Conclusion:* SFT appears to be a soft tissue tumour, that may arise in parosteal soft tissue and is capable of invading bone. The clinical behaviour as yet is not determined by histomorphology. In estimating behaviour one must rely on preoperative imaging (infiltration/metastases) and the complete pathologic work-up of the specimen.

**E118 Cyclooxygenase-2 Inhibitor for Pain Management in Osteoid Osteoma**

F. Boettner[1], R. Roedl[1], K. Wortler[2], C. Grethen[3], W. Winkelmann[1], N. Lindner[1]
*[1]Dept. of Orthopaedics, University Hospital of Muenster, Muenster, Germany, [2]Dept. of Radiology, Klinikum Rechts der Isar, Techn. Univ. Muenchen, Munich, Germany, [3]Dept. of Orthopaedic Surgery, Landeskrankenhaus Salzburg, Salzburg, Germany*

Thirteen patients with an osteoid osteoma were enrolled in a prospective trial to test whether rofecoxib, a selective cyclooxygenase-2 inhibitor, is as effective for pain control as acetylsalicylic acid. Each patient documented the pain level using a visual analog scale with 0 being no pain and 10 being unbearable pain during 2 days of no pain medication, 4 days of 500 mg acetylsalicylic acid three times a day, and 10 days of 25 mg rofecoxib once a day. Oral administration of 500 mg acetylsalicylic acid three times a day led to a significant decrease in pain at night, pain at rest, and pain induced by exercise. Twenty-five milligrams rofecoxib given once a day at midday showed the same remarkable improvement in pain at night, pain at rest, and pain induced by exercise. Rofecoxib in comparison with acetylsalicylic acid showed a trend toward lower pain levels in all categories. Rofecoxib offered a significantly better reduction in pain at rest during the day than acetylsalicylic acid. Results of the current study suggest that pain induction in osteoid osteoma is related to cyclooxygenase-2, an enzyme that is blocked by acetylsalicylic acid and rofecoxib. Conservative medical treatment with rofecoxib for osteoid osteoma is recommended whenever percutaneous intervention is associated with significant morbidity.

**E119 'Dedifferentiated' Adamantinoma of the Tibia: a New Entity**

H.M. Hazelbag[1], J.B. Laforga[2], H. Roels[3], P.C.W. Hogendoom[1]
*[1]Leiden University Medical Center, Leiden, The Netherlands, [2]Surgery Hospital Marina Alta, Denia (Allicante), Spain, [3]University Hospital, Gent, Belgium*

In adamantinoma of long bones, an osteofibrous dysplasia (OFD-) like form with only scattered epithelial elements and a classic form with abundant epithelial component are distinguished. OFD-like adamantinomas mainly occur in children and adolescents, behaving relatively benign. Classic adamantinomas predominate in adults and have a more aggressive clinical course. Because some OFD-like tumours have progressed to classic adamantinomas, it is currently accepted that the former is a potential precursor of the latter. Sarcomatous differentiation of the epithelial component in classic adamantinoma has hitherto been described in only one case, included in this report. We studied 3 male patients of 83, 63, and 42 years old. The first patient had sarcomatous dedifferentiation of the epithelial component in a large primary tibial adamantinoma. He underwent amputation and was well at latest follow-up at 23 months. In the second patient, sarcomatous dedifferentiation was shown in an en-bloc resected recurrence, 8 months after curretage. The third patient had had a radiologically slowly progressing osteolytic cortical tibial lesion since the age of 2, a pathological fracture at the age of 5 with subsequent anterior bowing, followed by en-bloc resection at the age of 37. This tumour showed classic adamantinoma. A local recurrence 3 years later showed a sarcomatously dedifferentiated epithelial component. The patient died of metastatic disease shortly thereafter. Histologically, the tumours showed loss of the original characteristic epithelial differentiation with transition to fields of highly polymorphic cells with strong nuclear pleomorphism, high mitotic count, and deposition of osteoid and chondroid matrix, mimicking osteosarcoma. However, the immunophenotype was identical to classic adamantinoma.
*Conclusion:* This new variant of adamantinoma shows that in addition to the mesenchymal-epithelial transformation in the early stage of development, progression to a more aggressive subtype may be associated with sarcomatous dedifferentiation (or 'transdifferentiation'), in which the epithelial immunophenotype is conserved.

**E120 Adenoviral Delivery of a Secreted form of Human Liver Carboxylesterase-2 Sensitizes Osteosarcoma to CPT-11**

M.A. Witlox, D. Oosterhoff, V.W. Van Beusechem, A. De gast, F.A. Kruyt, H.J. Haisma, G.R. Schaap, H. Bras, D.T. Curiel, H.M. Pinedo, W.R. Gerritsen, P.I.J.M. Wuisman
*VU University Medical Centre, Amsterdam, The Netherlands*

There are still too many patients who cannot benefit from current treatment modalities. Therefore, new therapeutic approaches are warranted. Adenoviral vector (Adv) based suicide gene therapy could be one such new therapeutic approach. CPT-11 (irinotecan) is an anticancer agent that can be considered as a prodrug, since it needs to be converted into SN-38 by the enzyme carboxylesterase (CE) to reach its full potency. SN-38 is 1000-fold more potent compared to CPT-11. We hypothesized that a secreted form of CE produced by tumour cells would diffuse through a tumour mass, leading to extracellular conversion of CPT-11 to SN-38. Once formed outside the cell, SN-38 should then more effectively kill untransduced, neighboring cells. We constructed a secreted form of human liver CE-2 (sCE2) by deleting of a microsomal retention signal. This sCE2 construct was subsequently cloned into a replication deficient adenoviral vector to produce Ad-sCE2. To study whether OS cells can be sensitized to CPT-11, four human OS cell lines (MG-63, MNNG-HOS, Cal-72 and SaOs-2) were infected with Ad-sCE2. The secreted enzyme was expressed by infected cells and converted CPT-11 to SN-38, thereby lowering the IC50 concentration for CPT-11 up to 1000 fold. Most importantly, primary cisplatin-resistant OS cells, derived directly from a patient, were sensitized 10 to 100 fold for CPT-11 by infection with Ad-sCE2. The expected bystander effect was confirmed by the observation that all tumour cells were effectively killed when only 1–10% of the cells were infected and a non-toxic concentration of CPT-11 was administered. Currently, experiments with human OS xenografts are being performed to assess the antitumour efficacy of Ad-sCE2 in combination with CPT-11 in vivo. In Conclusion, combined treatment of OS cells with Ad-sCE2 and CPT-11 had a prominent effect, even on chemotherapeutic resistant cells.

**E121 Role of Surgery in Local Treatment of Ewing's Sarcoma of the Extremities in Patients Undergoing Adiuvant and Neoadiuvant Chemotherapy: 20 Years Experience at Rizzoli Institute**

S.G. Giacomini[1], M.M. Mercuri[1], S.F. Ferrari[1], D.D. Donati[1], E.B. Barbieri[2], M.M. Manfrini[1], C.F. Forni[1], F.B. Bertoni[1], G.B. Bacci[1]
*[1]Istituti Ortopedici Rizzoli, Bologna, Italy, [2]Radiotherapy S. Orsola Hospital, Bologna, Italy*

*Background:* Although more and more patients with Ewing's sarcoma of bone (ESB) are being treated by surgery, the role of surgery and radiotherapy in the local treatment of this tumour has yet to be determined. In fact, because the outcome of ESB may differ according to the anatomical site of the tumour, Results reported in literature, which generally refer to series with tumours located in all sites, may be selection biased. Therefore we have retrospectively evaluated patients with ESB exclusively in the extremity and locally treated by surgery or radiotherapy.
*Methods:* 268 patients treated at Rizzoli from 1979 to 1996 for non-metastatic ESB of the extremities were assessed. Chemotherapy was administered according to four sequentially activated protocols. 136 patients were treated by surgery, 70 by surgery and radiotherapy and 60 patients by radiotherapy. Two patients underwent only chemotherapy.
*Results:* Follow-up ranged 5–23 years (mean=13years). 152 patients remained continuously free of disease, 108 relapsed, 2 died of chemotherapy toxicity and 6 developed a second malignancy. The 5-year event-free survival (EFS) and overall survival (OS) were respectively 62% and 69%. Although patients of all groups were matched for possible risk factors, the rates of 5-year event-free survival and local control were significantly lower in patients treated with radiotherapy compared to patients treated by surgery or surgery and radiotherapy (48% vs 66%, p=.002; 80% vs 94%, p=.0001). Furthermore, in Group 3 there were 6 secondary malignancies.
*Conclusion:* Our Results seem to indicate that surgery should always be considered in the local treatment of ESB of the extremities.

**E122 Evaluation of in vitro Effectiveness of Possible Candidates for Chemotherapeutic Treatment of Drug-Unresponsive Osteosarcoma Patients**

M Serra, G. Reverter-Branchat, M. Incaprera, K. Scotlandi, S. Perdichizzi, M.C. Manara, S. Benini, V. Cerisano, R. Strammiello, P. Piero
*Istituti Ortopedici Rizzoli, Bologna, Italy*

Development of drug resistance is the most important cause of failure of chemotherapy in high-grade osteosarcoma (OS). Therefore, treatments planned to circumvent drug resistance mechanisms may be the basis for therapeutic regimens aimed to increase the drug response rate of OS patients. The aim of this study was the evaluation of in vitro effectiveness of two drugs that can circumvent some of the most important mechanisms responsible for resistance to doxorubicin (PNU-159548) and methotrexate (trimetrexate). The in vitro cytotoxicity of these agents in combination with the drugs commonly used in OS chemotherapy were studied by using a panel of human OS cell lines resistant to doxorubicin, methotrexate and cisplatin, as well as a series of human OS cell lines established from surgical specimens of high-grade OS patients. The cells were simultaneously or sequentially exposed to PNU-159548 or trimetrexate in combination with doxorubicin, methotrexate, and cisplatin and the effect of each combination (additivity, synergism, or antagonism) was evaluated with the median effect plot analysis. The Results indicated that OS cells are, in general, sensitive to both PNU-159548 and trimetrexate. In particular, PNU-159548 resulted to be active also in OS drug-resistant cells, being effective against cell lines resistant to doxorubicin, methotrexate and, with a slightly lower efficiency, to cisplatin. The in vitro analysis of trimetrexate efficacy demonstrated a significant effectiveness in all drug-sensitive and drug-resistant OS cell lines analyzed, with the only exception of cell lines that developed resistance to MTX through increased levels of dihydrofolate reductase. Taken together, these data indicate that PNU-159548 and trimetrexate may be considered as promising candidates for planning new chemotherapeutic regimens to be addressed to high-grade OS patients who may be refractory to conventional treatments.

**E123 Genetic Aberrations Detected by Innovative Technologies in Methotrexate-Resistant Human Osteosarcoma Cell Lines**

C.M. Hattinger, M. Serra, G. Reverter Branchat, M. Incaprera, K. Scotlandi, S. Benini, M.C. Manara, P. Picci
*Istituti Ortopedici Rizzoli, Bologna, Italy*

Drug resistance is a multifactorial phenomenon mediated by the accumulation of genetic changes and therefore requires technologies that allow genetic analyses on a genome-wide scale. In this study, comparative genomic hybridization (CGH) on chromosomes and CGH on microarrays were used to identify only those genetic changes that were associated with development of resistance to methotrexate (MTX) in human osteosarcoma (OS) cells. Red-labeled reference DNAs from the parental drug-sensitive U-2OS and Saos human OS cell lines were co-hybridized with green-labeled test DNAs of two series of MTX-resistant variants selected by stepwise increasing in vitro concentrations of MTX. The direct comparison of MTX-resistant variants with their respective parental cell lines by CGH on chromosomes revealed that development of MTX resistance was associated with gain of the chromosomal regions 5q12-q15 and 11q14-qter in U-2OS variants and with gain of 8q22-qter in Saos-2 variants, suggesting the possible involvement of genes located in these regions. For the identification of amplified oncogenes in the MTX-resistant variants compared to their parental MTX-sensitive cell lines, CGH was performed on AmpliOnc microarrays (Vysis Inc) that contain oncogenes known to be frequently amplified in human cancers. In U-2OS MTX-resistant variants, amplification of MLL (11q23) and gain of FGR (1p36.2-p36.1) gene were consistently found, whereas Saos-2 MTX-resistant variants displayed gains of different genes depending on the level of resistance. Further analyses by molecular techniques detected amplification of the dihydrofolate reductase gene (5q11.2-q13.2), encoding for the target of MTX, in U-2OS but not in Saos-2 MTX-resistant variants. On the other hand, fluorescence in situ hybridization showed a relevant increase of C-MYC copy numbers only in Saos-2 variants. These Results suggest that genome-wide analyses of MTX-resistant OS cells by innovative technologies can be considered a useful tool for the identification of candidate genes that might be involved in MTX unresponsiveness.

**E124 Ewing Tumours of the Spine with Penetration into the Spinal Canal Results of the (EI)CESS Studies**

M. Kuhlen[1], S. Ahrens[1], M. Paulussen[1], A. Brentrup[2], A. Schuck[3], W. Winkelmann[4], H. Juergens[1]
*[1]University Children's Hospital, Muenster, Germany, [2]Dept. of Neurosurgery, Muenster, Germany, [3]Dept. of Radiotherapy, Muenster, Germany, [4]Dept. of Orthopedics, Muenster, Germany*

*Objectives:* Ewing tumours (ET) of the spine may be associated with cord compression. The prognostic impact of tumour penetration into the spinal canal was analysed in pts registered to the (EI)CESS studies.
*Patients:* Between 01/81 and 12/99, 116 pts with ET of the spine were registered in the (EI)CESS studies, representing 7.5% of the study population (1549 pts). At diagnosis 32/116 pts presented with tumour penetration into the spinal canal, 23/32 pts with localised disease, 9/32 pts with metastases to the lungs (6 pts), bone marrow (1 pt), skeletal system (1 pt) and lymph nodes (1 pt). For local therapy, 26/32 pts underwent primary surgery (laminectomy and/or tumour resection) at diagnosis. 4/32 pts underwent surgery after primary chemotherapy and 2/32 pts received definitive radiotherapy. 28/30 pts with surgery received postoperative radiotherapy. Surgery was intralesional in all pts, in 3/30 with intraoperative damage to the dura.
*Results:* To date 17/32 pts relapsed, 9/17 with local, 1/17 with combined, and 7/17 with systemic recurrences. After surgery, 2/2 pts presented with local recurrences, after surgery with radiotherapy, 6/14 with local, 1/14 with combined, and 7/14 with systemic recurrences (2/7 with primary lung metastases, 1/7 with primary bone marrow involvement). Of two pts with radiotherapy, one recurred locally. 3/4 pts with surgery after initial chemotherapy relapsed. 15/32 pts are in remission. EFS after 10 years was 44%. The principle relapse site was local recurrence (31%) compared to 8,9% in other primary sites.
*Conclusion:* Pts with ET of the spine with penetration into the spinal canal have an unfavourable prognosis due to failure of local control. The risk of dissemination into the spinal canal seems minor at least in pts without opening of the dura. Future treatment strategies must focus on better local control.

**E125 Bugged Out? Infection and Endoprosthetic Replacements**

L.M. Jeys, R. Grimer, S. Carter, R. Tillman
*Royal Orthopaedic Hospital, Holmfirth, United Kingdom*

*Introduction:* Endoprosthetic replacement (EPR) following Bone Tumour excision is common. A major complication of EPRs is infection, which can have disastrous consequences. This paper investigates the cause of infection, management and sequalae.
*Methods:* Over 10,000 patients have been treated over 34 years. Information collected includes demographic data, diagnosis, treatment (including adjuvant), complications, and outcomes. Data was analysed to identify any infection in EPRs, its management and outcome. Factors such as operating time, blood loss, adjuvant therapy, type of prosthesis (extendable or standard) were investigated. Outcomes of treatment options were evaluated.
*Results:* Data was analysed on 1265 patients undergoing EPR over 34 years. Giving a total follow up time of over 6500 patient years. 137 (10.8%) patients have been diagnosed with deep infection (defined by a positive culture [n=128] or a clinically infected prosthesis with pus in the EPR cavity [n=9]).49 (34%) required amputations for uncontrollable infection. The commonest organisms were Coagulase Negative Staphylococcus, Staphylococcus aureus and Group D Streptococci. The only satisfactory limb salvaging operation was 2 stage revision, which had 71% success in curing infection. Systemic antibiotics, antibiotic cement or beads and surgical debridement had little chance of curing infection. Infection rates were highest in the Tibial (23.1%) & Pelvic (22.9%) EPRs, ($p<0.0001$). Patients who had pre or post-operative radiotherapy had significantly higher rates of infection ($p<0.0001$), as did patients with extendable EPRs ($p=0.007$). Patients who had subsequently undergone patella resurfacing and rebushing also had a higher rate of infection ($p= 0.019$ & $p=0.052$).

*Conclusion:* Infection is a serious complication of EPRs. Treatment is difficult and prolonged. 2 stage revision is the only reliable method for limb salvage following deep infection. Prevention must be the key to reducing the incidence of this serious complication.

**E126 Histologic Grade Change in Locally Recurrent Soft Tissue Sarcomas**

P.C. Ferguson, A. Abudu, S.R. Carter, R.M. Tillman, R.J. Grimer, N. Deshmukh
*Royal Orthopaedic Hospital, Birmingham, United Kingdom*

*Introduction:* Soft tissue sarcomas (STS) have a reported local recurrence rate of between 5 and 30 percent. Recurrent tumours are often similar histologically to the initial tumour, however they are occasionally of higher histologic grade than the original lesion. Factors that predispose to this change in grade are not known. We sought to identify the frequency at which locally recurrent STS demonstrate a change in histologic grade, and to investigate the possible factors leading to this change. We also investigate whether a change in grade is associated with a poorer prognosis.

*Methods:* We identified 173 patients who developed locally recurrent STS, 116 of which had complete pathologic documentation who will form the basis of this study.

*Results:* Ninety-two patients (79%) had no change in histologic grade and 24 (21%) demonstrated an increase in histologic grade. The mean time to local recurrence was 21 months (range 2–171) in the unchanged group and 24 months in the changed group (range 2–69 months) ($p=0.56$). In the unchanged group 16% underwent radiotherapy and 17% chemotherapy, while 29% in the unchanged group underwent radiotherapy and none chemotherapy. Univariate analysis of time to local recurrence, histologic diagnosis and use of radiotherapy did not reveal significant differences between the groups who did and did not undergo change in grade. When the diagnosis of MFH was looked at separately, there was a trend toward a higher proportion in the group that underwent a grade change. Development of a change in grade was not associated with a poorer survival rate.

*Conclusion:* Increase in histologic grade occurs in approximately 20% of locally recurrent STS, but this phenomenon is not associated with a poorer prognosis than if the grade remains the same. We were unable to demonstrate any factors that could predict for a change in histologic grade.

